# A Q study: exploring the purpose of transdisciplinary dairy advisory services in Denmark

**DOI:** 10.1186/s13028-021-00610-1

**Published:** 2021-11-20

**Authors:** Wendy Holm, Dorte Bay Lastein

**Affiliations:** 1 LVK, Fynsvej 8, 9500 Hobro, Denmark; 2 Section for Animal Production, Department of Veterinary and Animal Sciences, Nutrition and Health, University of Copenhagen, Grønnegårdsvej 2, 1870 Frederiksberg C, Denmark

**Keywords:** Advisory network, Advisor style, Farmer demands, Farmer style, Knowledge-exchange, Q methodology, Teamwork

## Abstract

**Background:**

Structural changes in dairy farming increase farm complexity, thereby inducing a need to combine herd health management, technological solutions, legislation, and human relations among farmers, farm workers, and advisors. This complex situation may require ‘transdisciplinary advisory service’, i.e., a highly integrated network of both non-academic and different academic disciplines. While working in these networks, advisors need to offer specialized knowledge from their own field, interact in a dynamic relationship between different types of professions and facilitate complex processes. The objectives of this study were: (1) to identify and describe different advisor and farmer styles based on their reasons to engage in transdisciplinary advisory services at farm-level, (2) to identify any possible conflicting perspectives between advisors and farmers’ demand, and (3) to discuss these styles and conflicts in the context and future of advisory services for dairy herd health and production management.

**Results:**

Using Q methodology, we explored the purpose of transdisciplinary advisory service on dairy farms. The results were derived from correlations between 40 statements for 25 advisors and 33 statements for nine farmers. We identified three similar styles among advisors and farmers, characterized as: (1) *the teamwork and knowledge-focused style*, (2) *the production and economy-focused style*, and (3) *the economy and strategy-focused style*. These styles included reflections on financial aspects, production, knowledge-exchange and the teamwork process itself. In addition, different emphasis on animal welfare, farm strategy and follow-up procedures between the styles became evident.

**Conclusions:**

This Q-study suggests three comparable styles between advisors and farmers. The main differences between the styles related to the teamwork process and purpose, follow-up process, financial aspects, farm strategy, and operational production objectives. Therefore, styles and expectations should be explored and discussed to create a mutual understanding within a farmer-advisor(s)-team, and to clarify the farmer’s needs and demands, and how the advisors can best meet these expectations. This study illustrates the importance of exploring different advisor and farmer styles to get a mutual understanding of the purpose of the transdisciplinary collaboration.

## Background

The complexity of dairy farm systems in northern Europe is increasing due to constraints and requirement related to high production levels, growing herd sizes, lack of qualified labor and technology. Also, issues like animal welfare, food security, antimicrobial usage and sustainability affect the public acceptance of milk production. Such development is likely to result in increased requirements in terms of the competences of farm owners and workers and all other involved personnel, including adherent herd health and production advisors. This complex and dynamic situation could require change towards a coordinated and multifaceted, multi-stakeholder approach for the advisory services to guide and support an efficient production [1]. This approach can also be characterized as ‘transdisciplinary advisory service’. Transdisciplinarity has been defined as collaboration in highly integrated networks of both non-academic (i.e. farmers, farm workers, and technical advisors) and at least two different academic disciplines (i.e. agronomist, veterinarians) developing and working towards common goals [2].

When establishing a transdisciplinary network, it is important to get: “the right networks of actors together on the right things” [3]. While working in these networks, each advisor need to offer specialized knowledge from their own field and, at the same time, interact in the dynamic relationship between different types of professional experts [4], while keeping the specific farm in focus. This requires the ability to facilitate complex processes such as organizing the transdisciplinary working procedures toward articulated purposes, build trust in the network and manage collaboration conflicts, for instance due to diverging or competing goals [3], and facilitate open and honest dialogues [5] to avoid conflicts and maximize benefits. Hence, some advisors might move from a knowledge transferring expert style toward a more facilitating and communicative advisor style [6–10] or perhaps even develop the expertise to switch between advisory styles.

Farmer styles refer to how farmers’ position themselves in the advisory interaction, and intensive research has been conducted to conceptualize farmer styles and demands from various angles [8, 11, 12], and how different farmers information demands frames the configurations of advisory services and advisor styles within a country setting [11]. Danish farmer styles and veterinarian advisory styles were explored, and demonstrated diverging perception of value of individual advisory services [13]. Ingram [14] uncovered four principal farmer and, here agronomist, advisor styles reflecting the farmers’ and advisors’ position in the advisory interaction: (1) advisors behaving as classical proactive and powerful experts and farmers defering to their advices; (2) farmers’ being proactive and powerful and dictating the focus of the advicery service. Advisors are still perceived by the farmers as experts responding to the farmers’ request; (3) encounters between farmers and advisors are characterized by a poor understanding of the other parties knowledge and experience. Farmers utilize and modify advices to match their own understanding; (4) farmers and advisors combine their experience and knowledge and work together in a partnership.

With inspiration from Ingram [14], the present study focus on farmers’ and advisors’ styles related to the perceived purpose of transdisciplinary advisory service, as we emphasize the importance of clarifying and negotiating a shared vision [3]. In this study, we include veterinarians and other academic and non-academic dairy herd health management and production advisors under the common term “advisors”.

Using Q methodology, we investigated perceptions of the purpose of transdisciplinary advisory services among advisors and farmers by asking the participants*: What do you consider to be the purpose of transdisciplinary advisory services?* The specific objectives were: (1) to identify and describe different advisor and farmer styles based on their reasons to engage in transdisciplinary advisory services at farm-level, (2) to identify any possible conflicting perspectives between advisors (e.g., service suppliers) and farmers’ demand, and (3) to discuss these styles and conflicts in the context and future of advisory services for dairy herd health and production management.

## Methods

### The Danish dairy industry and advisory setting

In 2021, the Danish dairy industry consists of approximately 2500 fulltime specialized dairy farmers. The average age of the farmers is 57 years, and the average herd size is 250 cows plus young stock. Danish dairy farmers grow their own roughage (mainly grass and maize for silage). Most herds are localized in the western part of the country [15]. The specialized herd health and production advisory services in Denmark consists of private dairy-veterinarians working under compulsory herd agreements (a required number of annual visits depending on the herd sized) where legislation determines that animal welfare and biosecurity are the main focuses, and production secondary. Other advisors work either as consultants in smaller or larger private firms or, most commonly, as employed by large advisory cooperatives (e.g., owned by the farmers themselves). These advisors focus on roughage and feeding, production, economy and herd health management in their advisory service. Veterinarians and agronomists are mainly educated to become traditional experts in a ‘knowledge transferring’-style, but recently, topics like communication and qualitative methodology have also been introduced into the academic curriculum.

### Q methodology: a methodology to identify patterns of subjective perspectives

We used Q methodology to explore how Danish dairy farmers and specialized production advisors perceive the purpose of transdisciplinary advisory services. Q methodology is a mixed method approach that quantitatively condenses numerous qualitative views on a topic into a limited number of perspectives. The analysis is based on a very specific question (the Q-question): What do you consider to be the purpose of transdisciplinary advisory services?

**Fig. 1:**
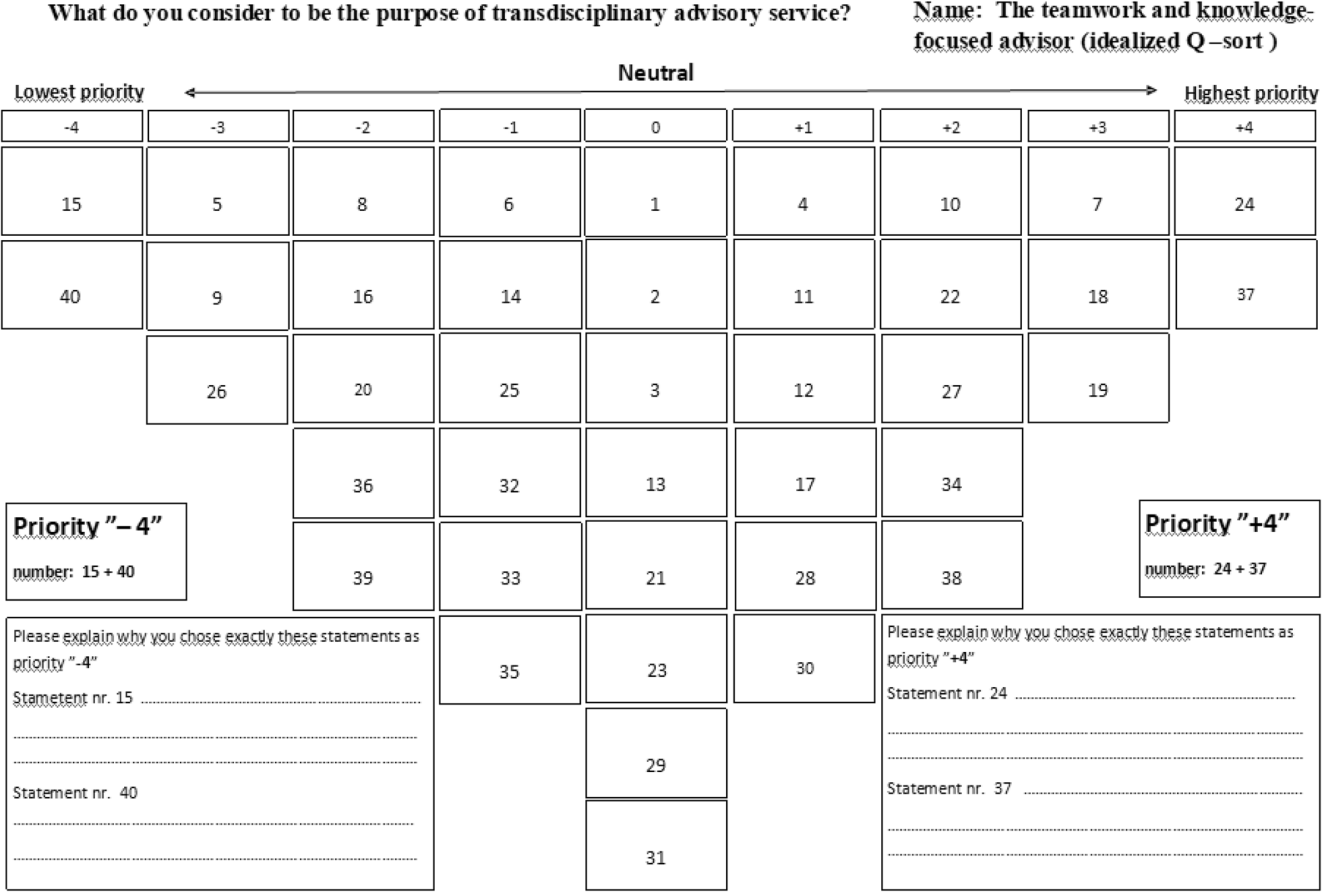
A Q-sort exemplified by an idealized Q-sort, i.e., how the typical advisor would sort the Q-set if he/she matched this style 100%. In an original Q-sort by an advisor belonging to this style, there would have been an elaboration of the reason for prioritizing statements 15, 40 lowest (disagree mostly) and statements 24 and 37 highest (agree mostly)

Q methodology consists of the following five steps (for further details consult textbooks on the subject, e.g. Watts and Stenner [16]):

1. *Selection of the P-set:* The P-set is a sample of respondents who are expected to have a clear and distinct view about the research question [17], i.e., a P-set is not supposed to be randomly selected. However, when it comes to viewpoints, maximum diversity is a goal for sampling (purposive sampling). The participants in this study were:
  1. Advisors (15 specialized dairy veterinarians from private practice and 10 other dairy consultants from private firms and advisory cooperatives) participating in a 2.5-year self-financed part-time postgraduate program. The program focused, among other things, on farmer and advisor styles and the interaction between advisors and the farmer in such knowledge-exchanging, transdisciplinary network. The 25 advisors were between 30 and 55 years old with a minimum of four years of experience in advisory services on dairy farms, and they worked in 22 different companies scattered all over the cow-dense Jutlandic region in Denmark.
  2. Nine dairy farmers participating in an educational program partially integrated with the advisor program mentioned above. The farmer’s educational program focused on farm strategy, and hence how to maximize the effect of the transdisciplinary advisory services provided by the 25 advisors. The farmers were invited to participate in the program by their bank and had agreed to implement the action plans developed in the context of the educational program. The farmers were between 28 and 53 years of age, and specialized farms had between 200 and 750 cows situated all over Jutland.
2. *Construction of the concourse:* A concourse is a technical and explicit construct covering ‘potentially everything about the topic’ [18], thereby including all possible statements a respondent could answer to the Q-question (i.e. all possible answers to the question ‘What do you consider to be the purpose of transdisciplinary advisory services?’). In this study, the concourse was constructed based on the authors’ experience of transdisciplinary advisory services, reflections on viewpoints in literature, interviews and discussions with dairy farmers and advisors over a period of 15 years. A concourse is potentially influenced by the authors’ preunderstanding of the subject; however, the concourse was discussed for further validation with peers who had experience in both dairy advisory service and Q methodology.
3. *Development of the Q-set:* We selected a subset of statements from the concourse to create what is known as a Q-set covering all the themes in the full concourse. Any single statement in the Q-set works as a potentially relevant answer to the question asked. We selected a Q-set that represented the breadth and depth of opinions in the concourse and only included statements contextually different from one another. Thirty-three statements were chosen for the final Q-set for farmers. The Q-set for advisors included the exact same 33 statements plus seven extra statements focusing on the advisors’ potential personal benefit from the transdisciplinary advisory service (i.e., 40 statements in total). Tables 1 and 2 include all statements. We then performed a pilot test to validate the Q-set, with three course leaders from the transdisciplinary education program as proxies.
4. *Q-Sorting:* On the first day of the educational programs, respondents (the P-sets, i.e., advisors and farmers) were asked to Q-sort (rank) the statements (Q-set) according to their own subjective opinion. The first day was chosen to minimize any bias due to preunderstandings.
  Each of the statements was printed on a separate card and marked with a random number for identification. Instructions on how to sort the statement cards were given in a short oral presentation immediately before the sorting, and each respondent also received a written step-by-step manual. The statement cards were sorted on A3-sized paper along a quasi-normal distribution (mean 0) ranging from ‘mostly agree’ (+ 4) to ‘mostly disagree’ (− 4). The participants could rearrange statement ratings under the entire process. The respondents were asked to elaborate on their reason for designating two statements as ‘mostly agree’ and two as ‘mostly disagree’. An example of a Q-sort is presented in Fig. 1.
5. *Q-factor analysis:* A factor analysis is a mathematical technique that reduces many individual variables (each respondent’s sorting of statements) into ‘families of sorts’. In the case of Q methodology, the factor analysis reduces a multidimensional cloud of data points into fewer distinguishable dimensions to identify patterns between the Q-sorts. The Q-sorts provided by advisors and farmers were analyzed separately. We used the PQMethod statistical program (available for free online [PQMethod, QMethod Page (schmolck.org)]) and followed the steps below, as described by Schmolck [19], Brown [20] and Webler et al. [14]:
  - *Data entry:* Every Q-sort was digitally reproduced as the randomly allocated number for each statement was typed manually into the software program in a quasi-normal distribution table identical to the original Q-sort. We controlled the validity of each sort twice before entering the next Q-sort.
  - *Unrotated factor analysis:* We used a Principal Component Analysis in PQMethod to examine the correlation matrix. The correlation between the Q-sorts was examined and eight factors were created automatically. The data file included eigenvalues for each factor and the accumulated variance of the data set; the higher numbers, the more explanatory power of the extracted factor.
  - *Factor rotation:* Factors were rotated analytically using the Varimax Rotation algorithm to optimize each factor and to ensure that every Q-sort (i.e., the sort of an individual advisor or farmer) was associated with a maximum of one factor. Each factor was based on the subjective perspective of a group of advisors or farmers, so a factor represents a “family of perspectives” and not just the view of a single advisor or farmer. To define the most appropriate number of factors for this data set, we first rotated two factors, then repeated the analysis for three, four, five and six factors, respectively.
  - *Flagging:* Factor loading values represent the degree to which a specific Q-sort, sorted by an individual advisor or farmer, correlates with a factor. Loadings can theoretically range from 1 (complete agreement) to -1 (complete disagreement). The final description of each factor was based on a weighted average of only those Q-sorts flagged as loading high on each specific factor. For this, we used the automatic pre-flagging function in the PQMethod program.
  - *Q-analyze:* The last analytic step in PQMethod produced an extensive report of various tables of factor loadings, factor scores for all statements (i.e., an average of the scores given to a specific statement by all Q-sorts associated with a factor), and consensus and distinguishing statements for each of the factors.
  - *Defining number of factors:* There is no objectively correct number of factors in Q methodology, so defining the most appropriate number of factors was an important part of the analysis. Inspired by Webler et al. [14], we analyzed the data with the following mindset: (1) *Simplicity:* To balance between a small number of factors making the viewpoints distinct and easy to understand, while not losing important and interesting information about the differences between advisors’ and farmers’ views on the topic; (2)* Distinctness:* Each factor had to include at least one high-ranked statement to distinguish it from the other factors; (3)* Stability:* We compared results using different numbers of factors to ascertain whether there was a group of advisors/farmers clustered in each group regardless of the number of factors. This would indicate that the individuals had similar views.
  - *Interpretation of the results and definition of perspectives:* Statements can rank high or low in one, two, or more factors. The highest-ranked statements in a factor defined the primary purposes of transdisciplinary advisory services for this particular advisor or farmer perspective. The lowest-ranked statements in a factor indicated purposes that were not prioritized as important for this perspective. The comments written by advisors and farmers during and after the Q-sorting (see above) helped us understand why participants either ‘mostly agreed’ or ‘mostly disagreed’ with a statement. We looked for the key elements of each perspective and weaved statements for each factor together into a narrative explanation of what each perspective considered to be the purpose of transdisciplinary advisory services. We paid particular attention to the list of statistically significant (P < 0.05) distinguishing statements (i.e., statements with high or low priority in only one factor). In addition, the high-ranked statistically significant (P < 0.05) consensus statements (i.e., statements ranked highly in all factors) were also taken into consideration.
  - *Comparing and naming the perspectives:* Once the perspectives were complete, we qualitatively examined the similarities and differences between them to validate that each perspective contributed a unique perspective to the overall understanding of the purpose of transdisciplinary advisory services on dairy farms. Hereafter, for explanatory purposes, we named each of the perspectives according to their most defining viewpoints and categorized them as advisor and farmer styles.

**Table 1:** Factor arrays for advisors

Number	Statement, advisors	Factor 1	Factor 2	Factor 3
1	To contribute positively to the net income of the farm	0	1	**4**^a^
2	To offer advice focusing on the economic consequences of current actions	0	2	**3**
3	To identify possible improvements in farm management	0	0	1
4	To provide a ‘whole farm’ advisory service	1	1	− 1
5	To educate the farm employees	− **3**	− **3**	− 2
6	To increase my own professional competencies	− 1	− 2	− 1
7	To ensure value in terms of the money invested by the farmer in transdisciplinary advisory services	3	2	2
8	To share the responsibility with the farmer	− 2	− 2	1
9	For people from my transdisciplinary network to recommend me as an advisor to other farmers	− **3**	− **4**	− 1
10	To improve the production results of the farm	2	**3**	3
11	To increase the impact of the advisory service when a team of advisors reach the same conclusion	1	− 1	1
12	To contribute with specialist knowledge from my own field of expertise	1	0	− 1
13	To contribute to the development of the farm strategy	0	0	0
14	To support the farmer to take more responsibility	− 1	− 2	2
15	To create extra revenue for myself	− **4**	− **4**	− **4**
16	To improve dairy herd health	− 2	**3**	− 1
17	To solve problems on the farm	1	2	1
18	To create results by working together	**3**	2	− 2
19	To help the farmer achieve the farm strategy	3	− 1	**4**
20	To educate the farmer in human resource management	− 2	− **3**	− **4**
21	To increase the farmer’s job satisfaction when cooperating with his/her advisors	0	1	2
22	To work with the farmer toward a common goal	2	3	0
23	To motivate the farmer to change farm management procedures	0	0	1
24	To benefit from the advisors’ different competencies	**4**	4	2
25	To expand my professional network	− 1	− **3**	− 2
26	To improve animal welfare	− **3**	2	− 1
27	To transfer knowledge from several professional perspectives	2	1	− 2
28	To cooperate on an equal footing with other professions	1	0	− **3**
29	To follow-up on agreed actions	0	0	0
30	To help the farmer gain an overview of priority action areas	1	1	0
31	To dare to raise any issue despite potential disagreement	0	− 1	0
32	To develop specific action plans in the transdisciplinary setting	− 1	0	0
33	To learn more about teamwork processes	− 1	− 2	− **3**
34	To support the farmer in reaching his/her goals	2	1	3
35	To support the farmer in his/her dialogue with the bank	− 1	− 1	1
36	To support the farmer in implementing standard operating procedures on the farm	− 2	− 1	0
37	To make use of all available knowledge regarding the interaction between feed and health	**4**	4	0
38	To provide the kind of advisory service that the farmer requests	2	0	2
39	To improve my own job satisfaction	− 2	− 2	− 2
40	To reduce the use of antibiotics	− **4**	− 1	− **3**

^a^Statements rated ‘mostly agree’ (+ 3 and + 4) and ‘mostly disagree’ (− 4 and − 3) are marked in bold

**Table 2:** Factor arrays for farmers

Number	Statement, farmers	Factor 1	Factor 2	Factor 3
1	To ensure value for the money in terms of my investment in transdisciplinary advisory services	**3**^a^	− 4	3
2	To obtain advice focusing on the economic consequences of current actions	2	3	2
3	To identify possible improvements in farm management	1	4	4
4	To improve animal welfare	− 2	− **4**	**3**
5	To obtain knowledge from several professional perspectives	0	**3**	0
6	To increase my job satisfaction when cooperating with my advisors	− **4**	− 3	− 2
7	To increase the net income of the farm	4	1	**4**
8	To ensure that my advisors share responsibility with me	− 2	0	0
9	To benefit from all available knowledge regarding the interaction between feed and health	0	4	1
10	To reduce the use of antibiotics	− **3**	− 2	− 1
11	To develop specific action plans in the transdisciplinary setting	1	− 2	− 1
12	To motivate me to change farm management procedures	− 1	0	− **3**
13	To ensure that my advisors contribute to the development of the farm strategy	− 1	− 2	0
14	To get support from my advisors to take more responsibility	− **4**	− **3**	− **4**
15	To receive a ‘whole farm’ advisory service	0	− 1	− 1
16	To help me educate my employees	− 1	− 2	− 2
17	To create results by working together	1	3	1
18	To follow-up on agreed actions	**4**	− **3**	0
19	To get help from my advisors to achieve the farm strategy	**3**	1	− 1
20	To help me improve human resource management at the farm	− 1	1	0
21	To work with my advisors toward a common goal	− 1	1	1
22	To benefit from my advisors’ different competencies	2	1	1
23	To solve problems on the farm	1	2	2
24	To improve dairy herd health	2	0	2
25	To improve the production results of the farm	3	1	**3**
26	To make it more likely that I will follow advice when my team of advisors reach the same conclusion	0	− 2	− **4**
27	To cooperate on an equal footing with my advisors	− 2	2	− **3**
28	To get support in reaching my goals	0	− 1	− 2
29	To gain an overview of priority action areas	1	2	− 1
30	To support me in implementing standard operating procedures on the farm	− 2	0	− 2
31	To receive exactly the kind of advisory service I request	− **3**	− 1	− **3**
32	That my advisors raise all necessary issues despite any potential disagreement	− **3**	− 1	2
33	To get support in my dialogue with the bank	2	0	1

^a^Statements rated ‘mostly agree’ (+ 3 and + 4) and ‘mostly disagree’ (− 4 and − 3) are marked in bold

## Results

Three factors (i.e., styles) were identified with the Q methodology for advisors and farmers, respectively. A fourth factor did not identify any distinctive statement based on the statistical evaluation. In addition, the overlap between factors became too large with four factors, resulting in more advisors and farmers belonging to two factors and thereby excluding them from the study results. Thus, two three-factor models were chosen. The analysis explained a total of 60% of the variance among advisors and 68% of the variance among farmers, indicating acceptable strength and explanatory power of the extracted factors, and sound and acceptable reductions of the correlation matrix [16]. Tables 1 and 2 show the full factor arrays for advisors and dairy farmers. It is important to note that the identical statements in the two factor arrays are numbered differently for the two analyses due to the different number of questions.

One advisor and one farmer style is presented as a long narrative description following the principles of Watts and Stenner [16]. Selected comments elaborating on the reasons for ‘mostly agree’ given during the Q-sort by advisors and farmers belonging to this style are included (*quotes are presented in italics*)*.* Interpretations of the remaining advisor and farmer styles are presented as shorter summaries. All interpretations focus on how the different styles position themselves in the advisory interaction and what topics the different styles prefer to work with.

Technical and statistical elements are presented at the end of the qualitative description of each style. Tables of statements with high priority (+ 3 and + 4) and selected distinguishing statements were created for all styles and is presented for one advisor style for demonstration purposes (Table 3). Notice that numbers and statements in Table 1 is identical to numbers and statements in Table 3. Interesting examples of similarities across all styles within each analysis are discussed after the description of all factors. Tables 4 and 5 present a short summary of the advisor and farmer styles, respectively, to clarify similarities and differences between the styles.

**Table 3:** Statements characterizing *the teamwork and knowledge-focused advisor style*

Highest priority statements (ranked 3 & 4)	To benefit from the advisors’ different competencies (24/4^a^) To make use of all available knowledge regarding the interaction between feed and health (37/4) To help the farmer achieve the farm strategy (19/3) To ensure value in terms of the money invested by the farmer in transdisciplinary advisory services (7/3) To create results by working together (18/3)
Lowest priority statements (ranked -3 & -4)	To create extra revenue for myself (15/−4) To reduce the use of antibiotics (40/−4) To improve animal welfare (26/−3) To educate the farm employees (5/−3) For people from my transdisciplinary network to recommend me as an advisor to other farmers (9/−3)
Selected distinguishing statements^b^	To help the farmer achieve the farm strategy (19/3) To transfer knowledge from several professional perspectives (27/2) To educate the farmer in human resource management (20/−3) To improve animal welfare (26/−3)

^a^statement number 24 was rated + 4

^b^Statements significantly different (P < 0.05) for *the teamwork and knowledge-focused advisor style* compared to the two other advisor styles

**Table 4:** Short summaries of the three advisor styles

Advisor styles	Teamwork and knowledge-focused	Production and economy-focused	Economy and strategy-focused
Purpose	Creating results by working together. The collaboration process is important in itself	Achieving specific production-oriented goals. Working together is a method to obtain better results, than one advisor could alone	Long term focus on strategy and economy. The collaboration process itself is less important
High priority statements, examples	To benefit from the advisors’ different competencies To create results by working together To ensure value in terms of the money invested by the farmer in transdisciplinary advisory services To help the farmer achieve the farm strategy	To make use of all available knowledge regarding the interaction between feed and health To improve the production results of the farm To improve dairy herd health To work with the farmer toward a common goal	To contribute positively to the net income of the farm To offer advice focusing on the economic consequences of current actions To support the farmer in reaching his/her goals To help the farmer achieve the farm strategy
High priority distinguishing statement, example	To transfer knowledge from several professional perspectives	To improve dairy herd health	To contribute positively to the net income of the farm
Low priority distinguishing statement, example	To improve animal welfare	To help the farmer achieve the farm strategy	To cooperate on an equal footing with other professions

**Table 5:** Short summaries of the three farmer styles

Farmer styles	Economy and strategy-focused	Teamwork and knowledge-focused	Production and economy-focused
Purpose	Prioritize financial aspects along with a long-term strategic focus Acknowledge the importance of follow-up, but beyond this, the collaboration process itself is less important	Creating results by working together in a network for knowledge exchange in order to identify potentials for improvements. The collaboration process is important in itself	Focus on operational production-oriented and financial aspects. Working together is a method to obtain better results, than one advisor could alone
High priority statements, examples	To increase the net income of the farm To follow-up on agreed actions To ensure value for the money in terms of my investment in transdisciplinary advisory services To get help from my advisors to achieve the farm strategy	To identify possible improvements To benefit from all available knowledge regarding the interaction between feed and health To obtain knowledge from several professional perspectives To create results by working together	To increase the net income of the farm To ensure value for the money in terms of my investment in transdisciplinary advisory services To improve animal welfare To improve the production results of the farm
High priority distinguishing statement, example	To follow-up on agreed actions	To benefit from all available knowledge regarding the interaction between feed and health	To improve animal welfare
Low priority distinguishing statement, example	That my advisors raise all necessary issues despite any potential disagreement	To ensure value for the money in terms of my investment in transdisciplinary advisory services	To motivate me to change farm management procedures

### Advisor styles

Details on advisors’ statements and rankings are given in the factor array in Table 1.

### Full interpretation of advisor factor 1: The teamwork and knowledge-focused advisor style

The first advisor style perceived it as the main purpose of transdisciplinary advisory service to create results by working together in a coordinated team effort. This effort should include the advisors’ different perspectives, competencies, and all available scientific knowledge regarding the interaction between feeding and health to achieve a goal (24/+4, 37/+4, 18/+3, 27/+2; i.e., statement number 24 was rated + 4 and so forth).

This advisor style seemed to value both collaboration within the advisory team (interdisciplinary) and collaboration between advisors and the farmer (transdisciplinary), perceiving the advisory service as a team effort through which advisors support the farmer to obtain results, reach goals and achieve the farm strategy (22/+2, 34/+2, 19/+3).

In addition, this advisor style was more interested in the short-term economic benefits (value for invested money, 7/+3) than in the long-term economic consequences of the advisory service, such as economic consequences of current action plans (2/0) or contributing positively to the net income of the farm (1/0). No personal benefits such as extra revenue (15/−4), job satisfaction (39/−2), increased competencies (6/−1), or expanded network (25/−1) were prioritized.

To some extent, this advisor style seemed to favor the collaboration process itself over more operational and measurable factors. For example, improving animal welfare (26/−3) and health (16/−2), and reducing the use of antibiotics (40/−4) were low-priority reasons to engage in transdisciplinary advisory service. Additionally, working systematically on farm processes to identify possible improvements on the farm (3/0), implementing standard operating procedures (36/−2), making practical oriented action plans (32/−1) and ensuring follow-up on agreed action plans (29/0) were not high priorities. The advisor style prioritized contributing to topics decided by the farmer (38/2) over topics with potential disagreement (31/0) or situations where the farmer needed motivation to change (23/0) indicating an eagerness to maintain a good relationship. In addition, education of farmers (20/−2) and farm workers (4/−3), and theoretical education in teamwork (33/−1) were not perceived as important purposes. Based on the above, this advisor style seemed to strive for a supportive advisory function, rather than a leading, challenging, inspirational or decision-making function. We named this style *the teamwork and knowledge-focused advisor style.*

Quotes:

‘We (advisors and the farmer) must collaborate to achieve the goal; using different competencies in a good collaboration process will increase the quality and value of the advisory service’ (advisor 3).

‘Transdisciplinary advisory services are all about creating synergy though collaboration’ (advisor 8)

In short, *the teamwork and knowledge-focused advisor style* focused on helping and supporting the farmer. Working together as a team and focusing on mutual knowledge-exchange while using the advisors’ different competencies seemed more important than contributing to the principal (organizational and financial) decision-making or influencing concrete detailed operational procedures on the farm. Our results indicate that *the teamwork and knowledge-focused advisor style* focused on short-term ‘value for money’ instead of the long-term economic benefits of the advisory effort. Based on the transdisciplinary teamwork and the advisors’ different scientific knowledge and competencies, *the teamwork and knowledge-focused advisor style* would often prioritize to help the farmer obtain results at farm level (the individual animal seemed to be unimportant), reach the farmers’ goals and fulfill the farm strategy.

Table 1 contain the full factor array (rating of all statements), Table 3 summarizes important viewpoints for *the teamwork and knowledge-focused advisor style* and Fig. 1 illustrates the idealized Q-sort, i.e. how a typical advisor belonging to this style would sort the Q-set if he matched this style 100% [16].

Technical and statistical details: *The teamwork and knowledge-focused advisor style* had an eigenvalue of 12 and explained 46% of the total study variance. Eight advisors loaded significantly on this factor. For this and other styles, it is important to be aware that the number of loadings on a factor is not representative of the distribution of viewpoints in the whole population as the P-set is not a random sample.

### Summary interpretation of advisor factor 2: The production and economy-focused advisor style

The second advisor style prioritized achieving results using the advisors’ different competencies and all available knowledge on the interaction between feeding and health (24/+4, 37/+4). Teamwork toward a common goal and improving dairy herd health, animal welfare and production results were perceived as important purposes of transdisciplinary advisory services (22/+3, 16/+3, 26/2, 10/+3), with high priority placed on obtaining specific, practically relevant, and clear operational objectives. Furthermore, the advisor style to some extend favor economic performance measures by rating value for invested money, economic consequences of current actions, and farm net income (gross margin) moderately high (2/+2, 7/+2, 1/+1). On the other hand, this style did not focus on strategy as a transdisciplinary purpose, neither helping the farmer to fulfill the existing farm strategy, nor contributing to develop a new strategy (19/−1, 13/0). Consult Table 1 for statements and rankings. We named this style *the production and economy-focused advisor style.*

In short, *the production and economy-focused advisor style* seemed to be interested in production results on an operational animal level. The responses indicated that this advisor style did not see farm strategy to be an important transdisciplinary purpose, nor did it perceive the collaboration process as a purpose in itself. Instead, a transdisciplinary teamwork could be seen as a measure to reach common goals and improve production results at farm and animal level, exemplified by the focus on specific operational objectives such as health and welfare.

Technical and statistical details: *The production an economy-focused advisor style* had an eigenvalue of 2 and explained 7% of the total study variance. Nine advisors loaded significantly on this factor.

### Summary interpretation of advisor factor 3: The economy and strategy-focused advisor style

The third advisor style focused on ‘hard values’ such as financial aspects (1/+4, 2/+3, 7/2), farm strategy (19/+4), and production results (10/+3), rather than ‘soft values’ like teamwork (28/−3, 18/−4), human resource management (20/−4), and animal welfare (26/−1). The only ‘soft value’ prioritized was to increase the farmers’ job satisfaction (21/+2). Helping the farmer to take responsibility in terms of reaching his/her goals (14/2, 34/3) and providing the kind of advisory service the farmers’ requested (38/2) were perceived as the purposes of transdisciplinary advisory services within this style. However, the advisor style did not value knowledge communicated from several professional perspectives (27/−2). We named this style *the economy and strategy-focused advisor styles.* Consult Table 1 for details on statements and rankings.

In short, *the economy and strategy-focused advisor style* focused on farm finances including the economic benefits of identifying and managing herd problems. The result point to an advisor style supporting farmers to reach their own goals and fulfill the existing strategy, rather than making new strategic decisions. This advisory style seemed to be based primarily on the services requested by the farmers. Teamwork and equality were given a low priority.

Technical and statistical details: *The economy and strategy-focused advisor style* had an eigenvalue of 2 and explained 6% of the study variance. Four advisors loaded significantly on this factor.

### Similarities and differences across all advisor styles

In the following section, we describe opinions common to all three advisor styles.

Improving production results and value for invested money (statement 7 and 10 were rated + 2 to + 3) were both prioritized as transdisciplinary purposes for all three advisor styles. Interestingly, education of farm employees (statement 5 rated from -2 to -3) was not perceived as an important transdisciplinary purpose for any of the advisor styles. In addition, ‘own benefit’, including creating extra revenue and improving job satisfaction (statement 15 rated -4, statement 39 rated -2) were not perceived as important purposes of advising in a transdisciplinary context.

Table 4 presents a short summary of each of the advisor styles to illustrate similarities and differences between the styles.

### Farmers styles

Details on farmers’ statements and rankings are given in the factor array in Table 2.

### *Full interpretation of farmer factor 1*: *The economy and strategy-focused farmer style*

The first farmer style prioritized financial aspects above all other matters (7/+4, 1/+3, 2/+2, 33/+2), and increasing the net income was the main economic driver of transdisciplinary advisory services (7/+4). To reach this goal, a high priority was set on advisors continuously following-up on agreed actions (18/+4), including agreements related to the existing farm strategy (19/3). However, this systematic approach did not include the operational level because tools to develop systematic work procedures were not prioritized (30/−2). At an operational level, increasing production (25/3) and promoting animal health were important purposes (24/+2), but were not linked to welfare issues (4/−2), nor to reducing the use of antibiotic substances (18/−3).

This farmer style represented an independent farmer (8/−2) who did not prioritize working equally toward common goals (17/1, 27/−2, 21/−1), nor making use of the advisors’ competencies when it comes to goal setting (13/−1). Advisors were expected to give all kinds of advice that they found relevant (31/−3), but this farmer style did not prioritize exploring differences of opinions with the advisors (32/−3). It seems likely that this was not about avoiding conflict, rather it was an expression of the low value placed on the potential benefit of obtaining knowledge from different professional perspectives (5/0). Based on their apparent self-confidence, farmers belonging to this style seemed to be convinced that they could make the best decisions alone based on facts and advice, so they did not need consensus among the advisors to reach a conclusion (26/0). We named this style *the economy and strategy-focused farmer style.* Consult Table 2 for details on statements and rankings.

Quote:

‘It is highly important that all actions focus on net income. Otherwise, there is no future’ (farmer 3)

‘Finances and net income are what the transdisciplinary advisory service is all about’ (farmer 4)

In short, *the economy and strategy-focused farmer style* focused on the strategic and economic purposes of the advisory services. Based on the responses, the farmer style appeared to be self-confident, independent, and focused on own goals and strategy. Advisors were perceived as sources of information or inspiration, but this farmer styles preferred to make the decisions themselves. *The economy and strategy-focused farmer style* expected intensive follow-up from the advisor, but otherwise did not value the cooperative process itself in any way.

Technical and statistical details: *The economy and strategy-focused farmer style* had an eigenvalue of 4 and explained 44% of the variation. Four dairy farmers loaded on this factor.

### Summary interpretation of farmer factor 2: The teamwork and knowledge-focused farmer style

The second farmer style valued equality and teamwork (17/3, 5/3) highly compared to the other farmer styles. Besides, this style prioritized knowledge sharing related to management, feed, and health, and seemed willing to change existing procedures to solve problems (3/+4, 17/+3, 29/+2, 23/+2). However, action plans, follow-up, and systematic operational procedures were not in focus (11/−2, 18/−3, 30/0). Advice focusing on the economic consequences of current actions was prioritized (2/+3), but other economic parameters were rated substantially lower compared to the two other farmer styles. (1/−4, 7/1). In addition, farm strategy (13/−2, 19/1) and animal welfare (4/−4) seemed to be unimportant. We named this style *the teamwork and knowledge-focused farmer style.* Consult Table 2 for details.

In short, *the teamwork and knowledge-focused farmer style* seemed to prioritize an advisory method, where farmer and advisors work together on an equal footing. Responses indicated that knowledge sharing regarding management, feed, and health were important purposes of the transdisciplinary advisory service, and this along with the teamwork process itself seemed to be more important purposes of the transdisciplinary advisory service than the farmers’ need to improve farm finances.

Technical and statistical details: *The teamwork and knowledge-focused farmer style* had an eigenvalue of 1 and explained 13% of the variation. Two dairy farmers loaded on this factor.

### Summary interpretation of farmer factor 3: The production and economy-focused farmer style

The third farmer style presented a clear focus on operational production, as animal welfare (4/3) and herd health (24/2) were prioritized as means toward improving production (3/+4, 25/+3) and finances (7/+4, 1/+3, 2/+2). The farmer style represents an independent farmer who does not value working as an equal team member toward a common goal (17/+1, 27/−3, 21/+1, 22/+1). Instead, advisors were expected to give relevant advice (31/−3), also broach issues of expected disagreement (32/2) to solve problems (23/2). Farmers belonging to this style preferred to discuss issues rather than seek agreement with their advisors (26/−4), while not prioritizing the implementation of standard operating procedures, nor valuing follow-up or the creation of an overview of important areas for action (20/−1, 18/0, 29/−1). Therefore, the advisors’ professional knowledge was valued as an immediate source of information (i.e., classic knowledge-transferring role), rather than having the advisors as partners in an ongoing supportive and motivational process (28/−2, 12/−3). We named this style *the production and economy-focused farmer style.*

In short, *the production and economy-focused farmer style* perceived transdisciplinary advisory services as a way to gain knowledge from several advisor perspectives and to engage in dialogue with the advisors. Farmers belonging to this style are open to being challenged on their beliefs to improve animal welfare, health, production, and farm finances. *The production and economy-focused farmer style* seemed more likely to focus on operational animal parameters (e.g., welfare and health) than on planning and follow-up procedures.

Technical and statistical details: *The production and economy-focused farmer style* had an eigenvalue of 1 and explained 11% of the variation. Three dairy farmers loaded on this factor.

### Similarities and differences across all farmer styles

Advice focusing on the economic consequences of current actions (statement 2 ranked from + 2 to + 3), production results (statement 25 ranked from + 1 to + 3) and solving problems (statement 23 ranked from + 1 to + 2) were prioritized as somewhat important purposes of transdisciplinary advisory services for all three farmer styles.

Furthermore, there was agreement across all three farmer styles that advisors should not always deliver the services that farmers ask for (statement 32 ranked from -1 to -3). One statement that all farmer styles clearly disagreed with was having ‘support in taking more responsibility than you do today’ (statement 14 rated -3 to -4), indicating that farmers already carry the full operational and economic responsibility.

Table 5 presents a short summary of each of the farmer styles to illustrate similarities and differences between the styles.

## Discussion

### Choice of the Q methodology

This study is based on Q methodology, which is a mixed method approach using a quantitative methodology (factor analysis) to classify a spectrum of qualitative responses to a specific question into clusters [16]. Q methodology was chosen for two reasons:

1. By exploring how participants ranked opinion statements, the method revealed the subjective patterns of perspectives on transdisciplinary advisory services among the groups of respondents (advisors and farmers).
2. The participants’ perspectives could directly and quantitatively be categorized in a consistent manner, as everyone sorted the same set of statements [21].

Using this mixed method, we were able to quantitatively cluster opinions and thereby qualitatively describe different subjective approaches to transdisciplinary advisory services among specialized dairy farmers and specialized advisors. Compared to other purely qualitative methods, Q methodology allowed us to include more people within a shorter timeframe than would have been possible in classic, semi-structured interviews followed by a full transcription and an inductive and/or abductive analysis of empirical material, for example based on grounded theory [22]. Q methodology also allowed a deeper understanding of the studied phenomenon and validation of the results (e.g., written argumentation of high- and low-priority statements on the scorecard, not presented) compared to more quantitative approaches i.e., anonymous questionnaires answered by a larger population. In a sequential questionnaire, the respondents may change their mind about the early questions once they have reflected on the final questions. The Q-sort requires the respondent to assess all statements at the same time and move them around until the ranking is perceived as satisfactory for the individual participant. Thus, the sorting process in the Q-procedure allowed participants to rank and re-rank all statements providing insight into a reflected and detailed picture of their perceptions.

### Sampling and potential biases

In this study, we included data from specialized advisors and farmers. Farmers were younger than the average Danish dairy farm owner, and only one farm was smaller than the Danish average dairy herd-size. Farmers were invited by their banks to participate in the time-consuming and expensive program, only conventional farmers were enrolled. Probably, there is a bias towards farms and farmers that the banks considered to be part of the near future. It is also reasonable to assume that banks and farmers wanted to gain value from their investment, be it money or knowledge. Interestingly, only one consensus statement relating to finance was identified among the farmers, meaning that not all the farmers in the study had economy as their main focus. As the farmers agreed to be in the program, perhaps a bias towards positive acceptance of advisory services in general could be present.

Likewise, the program for advisors was time-consuming and expensive, and most of the attending advisors were in the middle of their professional carrier. Again, we assume that these advisors in general were positively interested in transdisciplinary advisory service. As the participants were not randomly included in the study, we might have missed an unknown number of styles. However, the aim of Q methodology is to categorize and explore the depth of different viewpoints (we call them styles) within a group of people, not to quantify proportions of individuals within a style nor to identify all possible styles. We are aware that selection bias could impair the results and we emphasize the importance of not blindly extrapolating the concrete results (i.e., the identified total six advisor and farmer styles) into other contextual settings. Instead, we encourage to use the study conclusion (i.e., that a variety of styles exit) to acknowledge the need for exploring and understanding the existence of different farmer and advisor styles when entering a transdisciplinary collaboration.

An inadequate concourse and Q-set are possible risks embedded in the study design, if they do not represent the full spectrum of possible purposes of transdisciplinary advisory services on dairy farms (i.e., lack of saturation). We have tried to control these risks by consulting experienced peers during the construction of the concourse and the Q-set. Also, misinterpretation of unclearly written statements during Q-sorting (classification bias) could happen. That is why, we did a pilot study to control the readability of the Q-set and test our spoken introduction of the sorting process.

The participants could have introduced a bias if they have missed the transdisciplinary dimension of the question and instead focused on advisory service in general. We have tried to control this bias by clarifying right before the Q-sorting, that focus should be directed exclusively on transdisciplinary advisory service. Despite this clarification, a possible risk exits, that some participants could have focused on ‘monodisciplinary’ advisory service instead when sorting the Q-set. The respondents were asked to elaborate on their reason for designating two statements as ‘mostly agree’ and two as ‘mostly disagree’, and these elaborations all point toward a transdisciplinary focus. Though, it might be possible that some Q-sorts and thereby elements of the styles would be representative for participation in both transdisciplinary and monodisciplinary advisory settings.

### Farmer and advisor styles in a transdisciplinary advisory setting

Once we had analyzed all the perspectives, we named each perspective according to the style. The same three styles were identified for advisors and farmers: *Teamwork and knowledge; Production and economy; Economy and strategy*. It is important to note that these names are simplified structures, and that each style draws from more than two areas of interest.

Partnerships, where farmers and advisors work together and combine their knowledge and experience, have been demonstrated to “provide the right context for effective knowledge exchange” [14]. For *the teamwork and knowledge-focused advisor style* and *the teamwork and knowledge-focused farmer style*, the transdisciplinary advisory process itself seemed to be important. The other farmer styles perceived the transdisciplinary advisory service as a way to gain better results compared to situations with only one advisor. Differences within and between advisor and farmer styles in a transdisciplinary team could potentially cause tensions in the collaboration. This distinction between ‘process’ and ‘result’ as a purpose seemed to be an important difference between the styles and should be part of an alignment of expectations when initiating a transdisciplinary collaboration.

The importance of follow-up varied widely across the farmers’ styles. In contrast, advisors rated follow-up as neutral. There are several possible definitions of ‘follow-up’; (1) repeated herd visits and intensive quantitative monitoring [23]; (2) evaluation of prospective planned herd-specific interventions in trial set ups [24], and; (3) varying qualitative exploration with feedback, ongoing dialogue, and networking involving participants both at the farm and externally [25–27]. Regardless of the definition, our study gives emphasis to the importance of discussing the level and method of follow-up and the areas of responsibility before initiating transdisciplinary teamwork. To meet the expectations of those like *the economy and strategy-focused farmer style*, advisors should put more effort and time into follow-up procedures. When working with a *teamwork and knowledge-focused farmer style*, the advisor might need to overcome the farmer’s reluctance to follow-up by facilitating the process or including the follow-up as an integrated aspect of the advisory service.

All advisor styles and two of the farmer styles prioritized ‘value for money invested in transdisciplinary advisory service’ as being very important, but *the teamwork and knowledge-focused farmer style* did not perceive value for money to be interesting. Instead, this farmer style focused on knowledge sharing and teamwork. It is therefore important that advisors do not assume that all farmers will be motivated by financial incentives, as previously studied [10, 13, 28].

Increased job satisfaction was not perceived to be a purpose of transdisciplinary collaboration for any of the farmer styles (consensus statement, low priority). However, *the economy and strategy-focused advisor style* prioritized increasing the farmer’s job satisfaction through financial or production improvements. The aim of increasing the farmer’s job satisfaction is a natural human social motive, because work is not just about increasing personal joy and happiness, it also involves helping other people to find theirs [29].

Interestingly, there was considerable variation in how animal welfare as a transdisciplinary topic was prioritized across both advisor and farmer styles. *The production and economy-focused advisor style* and *the production and economy-focused farmer style* prioritized welfare as being important. *The teamwork and knowledge-focused advisor* and *the teamwork and knowledge-focused farmer style*, on the other hand, rated welfare low in the transdisciplinary setting. Based on comments made on the scorecard by some participants after the Q-sort, welfare was not necessarily associated with the transdisciplinary advisory setting.

As dairy farms around the world expand, farmers are becoming increasingly reliant on hired employees for milking and performing other daily management procedures. Therefore, human resource management (HRM) has become a principal function of many farms [30]. Helping farmers to improve HRM was given a considerably low priority across all three advisor styles in this study, yet this could be an area of potential improvement on many farms. If advisors in the transdisciplinary setting had the competence and willingness to help the farmer with HRM, it might be possible to eliminate some of the high turnover of employees that has traditionally characterized dairy farms [27].

*The teamwork and knowledge-focused advisor style* and *the economy and strategy-focused advisor style* prioritized offering exactly the kind of service the farmer requested. However, *the teamwork and knowledge-focused farmer style* and *the production and economy-focused farmer style* did not want to restrict the advisors to the requested area of advice giving. Rather, they preferred the advisor to proactively broach any topics, reflections, experiences, and knowledge that they considered relevant to the farm. Such an approach, where farmers expect the advisors to challenge them more strongly in the knowledge exchange encounter has previously been demonstrated [8, 14]. In order to meet such farmer demand, advisors must have both professional knowledge and personal competences [11]. *The production and economy-focused farmer style* even placed importance on their advisors broaching issues of expected disagreement, indicating that an interactive dialogue-based approach from the advisors would be appreciated by some farmers. This finding is supported by a study from UK, in which the majority of farmers valued discussions with their veterinarians, while only a small proportion of veterinarians actively used discussions during farm visits [30].

*The economy and strategy-focused advisor style* prioritized a supportive advisor role based on the farmer’s goals and requests. *The production and economy-focused advisor style,* on the other hand, prioritized collaborating with the farmer towards a common goal. Whether the transdisciplinary team works toward the farmers’ goals or a common goal, advisors should explore the farmers’ expectations in order to choose a suitable communication strategy to support their advice, as described in various other contexts [31–34].

Different farmer styles have different demands, which raises at least two important questions:

1. How can advisors provide an advisory service suitable to meet different farmer styles’ needs and demands?
  Klerkx et al. [11] have proposed a model for ‘best fit’ of advisory service for a particular type of farmer in a pluralistic advisory system. (1) An inter-organizational system of service supply with collaboration between suppliers. Participating advisory organizations both cooperate and compete; (2) Organized expert teams. Generalists have first-line contact with the farmers and, when needed, they can bring in expertise from the expert teams; (3) Systems of private and public cooperation can work together on topics that require a long-term perspective and are difficult to turn into a commercial service.
  Most of the model presented by Klerkx et al. [11] have been formally established in Denmark. However, in our point of view, the model is not implemented effectively in everyday life among advisory service suppliers. Probably, advisors are primarily responsible for this somewhat in-effective situation due to lack of trust in each other, fear of losing income, fear of being replaced by more capable advisors etc. These issues might be even more dominant in transdisciplinary advisory situations, where advisors need to work beyond their professional comfort zone. Further research including action research to overcome such sociological issues could be conducted in the future.
  In Denmark, a farmer chooses the advisors he prefers, for example a veterinarian from a private practice and a dairy consultant from a private firm or an advisory cooperative. These advisors need to learn how to collaborate in a transdisciplinary setting, sometimes without knowing or even trusting each other. Thus, the ability to facilitate open and honest dialogues to avoid conflicts [5], build trust in the network and manage the conflicts that might arise in the collaboration [3] seems important.
  From a future perspective, the Danish ‘choose your own advisors’-model might need to be revisited to provide the opportunity to create a ‘better fit’ between farmer demand and advisory supply in general and especially within a transdisciplinary setting.
2. Do advisors need to be generalists or specialists?
  There could be a need for both, and many advisors need to balance themselves between specialization and universality [11]. Complementary advisor networks could be a way to facilitate knowledge-exchange between advisors [4, 35]. Besides, in such a network, the advisors will get to know specialists from other professional disciplines providing each advisor with the opportunity to help the farmer gain access to such expertise, when specialized knowledge is required [11]. For instance, the educational program behind this study included four different kinds of networks: A transdisciplinary network including all participants, several small transdisciplinary networks working on each farm, an interdisciplinary network for education and knowledge-exchange for the participating advisors, and likewise for the participating farmers.

### Implications for transdisciplinary advisory services in the future

This study allowed us to explore the possibilities and barriers in the beginning of a collaborative process, with the hope that any self-reflection during the sorting process could increase the likelihood of establishing a successful transdisciplinary collaboration. Our results can be used to support anyone interested in transdisciplinary advisory services with the idea that different perspectives on the same phenomenon can exist and be an obstacle for successful collaboration. Such misunderstandings between advisors and farmers have previously been identified across production types and nationalities [14, 22, 23].

This study has been about ‘why’ to engage in transdisciplinary advisory service. A logical next step would be to explore ‘how’ to succeed on the operational level. Facilitation as an advisory style has been used in Denmark in the organic dairy sector with some success [36]. Future studies could examine relevant training programs to establish a shared understanding of transdisciplinary facilitation methods and networks and how this could be operational in every day advisory service. Here, assembling the transdisciplinary teams based on farmer and advisor styles would be an interesting study in order to meet the individual farmer’s demand. Also, it would be interesting to explore, if the transdisciplinary approach can release the expected (economic) potentials.

## Conclusions

This Q-study suggests three similar styles among advisors and farmers: (1) *the teamwork and knowledge-focused style*, (2) *the production and economy-focused style*, and (3) *the economy and strategy-focused style*. The main differences were related to financial aspects, farm strategy, operational production objectives and the follow-up process. The teamwork process could be a purpose itself, or the method used to provide results. In addition, advisors could either perceive the advisory process as a supportive and informative function based on the farmer’s goals and requests, or an advisory process primarily intended to challenge the present situation. This study indicated that different farmer and advisor styles probably exist within most transdisciplinary settings. Therefore, styles and expectations should be explored and discussed to create a mutual understanding in the team, and to clarify farmer demands and how the advisors can meet these expectations. The identified styles from this study could be used as guidelines.

## Data Availability

The datasets used and/or analyzed during the current study are available from the corresponding author on reasonable request.

## Declarations

### Prior publication

Data have not been published previously.
